# Verticillium Wilt of Olive and Its Control: What Did We Learn during the Last Decade?

**DOI:** 10.3390/plants9060735

**Published:** 2020-06-11

**Authors:** Nuria Montes-Osuna, Jesús Mercado-Blanco

**Affiliations:** Departamento de Protección de Cultivos, Instituto de Agricultura Sostenible (IAS), Agencia Estatal Consejo Superior de Investigaciones Científicas (CSIC), Avenida Menéndez Pidal s/n, Campus “Alameda del Obispo”, 14004 Córdoba, Spain; nuriamontes@ias.csic.es

**Keywords:** biological control agents, breeding for resistance, defoliating and non-defoliating, integrated disease management, *Olea europaea*, organic amendments, pathogen detection, soil microbiota, vascular disease, *Verticillium dahliae*

## Abstract

Verticillium (*Verticillium dahliae* Kleb.) wilt is one of the most devastating diseases affecting olive (*Olea europaea* L. subsp. *europaea* var. *europae*a) cultivation. Its effective control strongly relies on integrated management strategies. Olive cultivation systems are experiencing important changes (e.g., high-density orchards, etc.) aiming at improving productivity. The impact of these changes on soil biology and the incidence/severity of olive pests and diseases has not yet been sufficiently evaluated. A comprehensive understanding of the biology of the pathogen and its populations, the epidemiological factors contributing to exacerbating the disease, the underlying mechanisms of tolerance/resistance, and the involvement of the olive-associated microbiota in the tree’s health is needed. This knowledge will be instrumental to developing more effective control measures to confront the disease in regions where the pathogen is present, or to exclude it from *V. dahliae*-free areas. This review compiles the most recent advances achieved to understand the olive–*V. dahliae* interaction as well as measures to control the disease. Aspects such as the molecular basis of the host–pathogen interaction, the identification of new biocontrol agents, the implementation of “-omics” approaches to unravel the basis of disease tolerance, and the utilization of remote sensing technology for the early detection of pathogen attacks are highlighted.

## 1. Introduction

Olive (*Olea europaea* L. subsp. *europaea* var. *europaea*) is cultivated between latitudes 30^°^ and 45^°^ in Mediterranean-type climate regions of both hemispheres, a tree crop integrating an unique set of morphological and developmental characteristics suited to the relatively dry, rustic conditions of these geographical areas [1,2]. Olive is the most iconic tree in the Mediterranean Basin, with huge economic, social, and ecological importance [3]. The multiple uses of cultivated and wild (*O. europaea* L. subsp. *europaea var. sylvestris* Brot.) olives as a source of food, wood, and cattle fodder explain the spread of olive groves with the expansion of human civilization [2]. Approximately 10.5 million ha are currently devoted to olive cultivation around the world, the Mediterranean Basin accounting for 98% of this surface [3]. Spain is the leading producing country of olive oil and table olives, Tunisia ranks second, followed by Italy, Turkey, and Greece (data from the 2017/2018 cropping season available at the International Olive Council database, [4]). Andalusia region, at the southernmost part of the Iberian Peninsula, concentrates the largest cultivation area of this tree crop [5].

**Figure 1:**
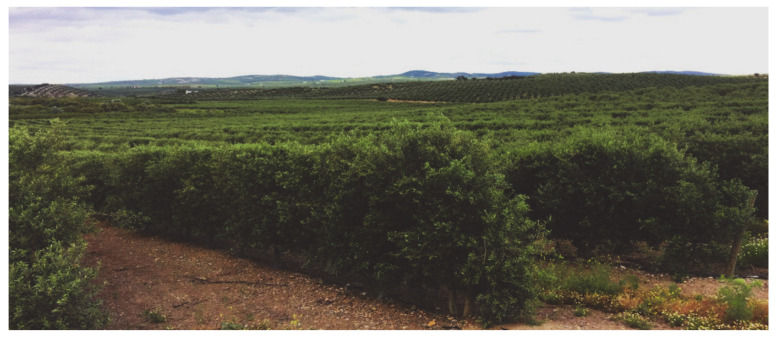
A super high-density hedgerow olive orchard located in Córdoba province (Spain).

During the last two decades, changes introduced in modern olive cultivation systems have revolutionized this crop, mostly aiming to increase yield [6,7] and production [8,9] and to improve management and mechanization practices [10]. For instance, the development of super high-density hedgerow orchards [11] (Figure 1) and the use of drip irrigation systems are modifying the traditional olive landscape built over millennia in the Mediterranean countries. While these benefits are desirable, the increasing reduction in the number of cultivars or practices such as high inputs of fertilizers or fungicides may have detrimental consequences, namely the reduction in olive genetic diversity or harmful effects on the soil microbiota [12,13]. These alterations, coupled with the high vegetation densities usually present in high-density orchards, may pose problems related to increasing the incidence and severity of specific olive pests and soil-borne diseases, yet have been insufficiently evaluated [10,14]. 

**Figure 2:**
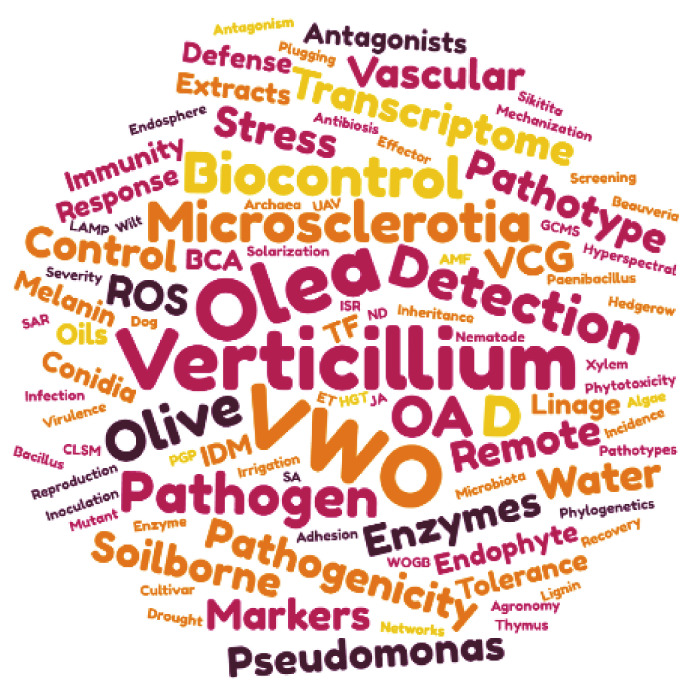
Tag-cloud showing the most relevant keywords cited in the bibliography consulted to produce this review article. The figure was built using a free online word cloud generator [20]. The importance of each tag is visually weighted according to its frequency of use. The acronyms used are defined as follows: Arbuscular Mycorrhizal Fungi (AMF), Biocontrol Agents (BCA), Confocal Laser Scanning Microscopy (CLSM), Defoliating (D), Essential Oils (oils), Ethylene (ET), Gas Chromatography–Mass Spectrometry (GCMS), Horizontal Gene Transfer (HGT), Induced Systemic Resistance (ISR), Integrated Disease Management (IDM), Jasmonic Acid (JA), Loop-Mediated Isothermal Amplification (LAMP), Non-Defoliating (ND), Organic Amendments (OA), Plants Extracts (extracts), Plant Growth Promotion (PGP), Reactive Oxygen Species (ROS), Salicylic Acid (SA), Systemic Acquired Resistance (SAR), Transcription Factor (TF), Unmanned Aerial Vehicle (UAV), Vegetative Compatibility Groups (VCG), Verticillium Wilt of Olive (VWO), Water Disinfestation (Water), World Olive Germplasm Bank (WOGB).

Currently, Verticillium wilt of olive (VWO), caused by the hemibiotrophic soil-borne fungus *Verticillium dahliae* Kleb., is considered one of the most devastating olive diseases and a major limiting factor for olive oil production. Besides economic losses due to tree mortality and fruit yield reduction, the negative effect on the commercial value of virgin olive oil has been recently demonstrated because of the poor organoleptic properties from fruits of *V. dahliae*-infected trees [15]. The aim of this review is to compile and discuss the most recent advances to enhance our knowledge of the olive–*V. dahliae* pathosystem and the efforts to control the spread and effects of the disease. We will focus on the literature produced during the last ten years (Figure 2). For general aspects related to the biology and genetics of the pathogen, epidemiological factors contributing to the expansion of the disease, and the strategies for its control, interested readers are kindly invited to consult the earlier comprehensive reviews by López-Escudero and Mercado-Blanco, Tsror, Jiménez-Díaz and co-workers, and Mercado-Blanco and López-Escudero [16,17,18,19]. 

## 2. Modern Olive Cropping Systems and Verticillium Wilt: Finding the Balance between Management Practices and Disease Risk

**Figure 3:**
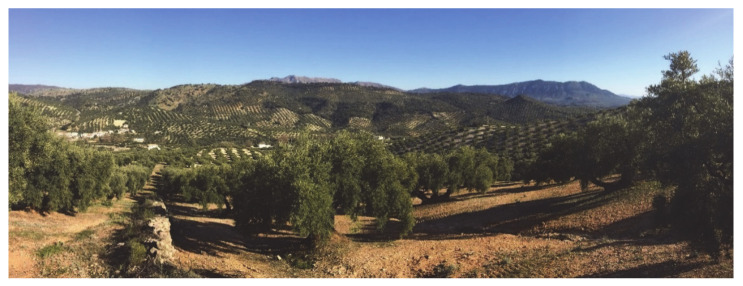
The traditional olive trees landscape in the Mediterranean Basin. Olive cultivation is part of the history, culture, landscape, and economy of this region. It is a tree crop well adapted to Mediterranean climatic conditions.

Over centuries, the traditional olive landscape was shaped under the low precipitation irregular rain regimes usually found in Mediterranean-type climatic conditions. During the last couple of decades, however, olive cultivation systems have experienced significant changes aiming to increase productivity and facilitate mechanization [11]. This has led to a different concept of the olive orchard coupled with more efficient management practices. Thus, the traditional low-tree-density olive orchard managed under rainfed conditions (Figure 3) is being replaced in some areas by high-density (250-400 trees/ha) or super high-density hedgerow orchards (1500-2200 trees/ha), (Figure 1) along with highly efficient drip irrigation systems [11].

However, the transition to modern olive cropping systems may pose risks such as the increased incidence and severity of VWO. Moreover, inadequate agronomic practices can increase the dispersion of the pathogen, seriously compromising olive production in many growing areas. On one side, this potentially enhanced exposure to VWO (and other traditional or emergent pests and diseases) may relate to new planting densities and frames in soils where the disease/pathogen has been already present, a scenario that is often overlooked from the phytopathological perspective. On the other hand, it is already known that in many cases the onset of the disease coincides with the reconversion of olive orchards from dry land to irrigation [21,22]. Two explanations have been proposed, both supported by experimental evidence. One is based on assays performed with susceptible olive varieties under controlled and field conditions, which have demonstrated that highly irrigated plots showed significantly higher VWO incidence than those subjected to reduced irrigation doses [22,23,24,25]. Moreover, the development of disease incidence may be influenced by the number of years since irrigation was implemented [22,23]. The second explanation relies on reports suggesting that the disease development is not directly influenced by the irrigation frequency but by the water content in the soil profile. Indeed, keeping a satisfactory water content level below 24% may delay or slow down the VWO onset/development [24]. It is worth mentioning that a high percentage of visited olive orchards (93.1%) in these studies were planted with the susceptible cultivars Picual and/or Hojiblanca [22], both of them very susceptible to *V. dahliae* attacks [16]. “Picual” and “Hojiblanca” are commonly used for olive oil production due to their high productivity and climatic adaptation. The planting of new varieties, such as “Arbequina” (moderately susceptible) and “Frantoio” (tolerant) may decrease the risk of pathogen dispersion, since irrigation frequency did not seem to influence the disease progress and low disease rates have been reported for both cultivars [23].

## 3. Knowing the Enemy and Its Most Dangerous Representative for Olive: The Defoliating Isolates of *Verticillium dahliae*

The reproduction of *V. dahliae* is strictly asexual. Therefore, the only possible way to exchange genetic material among its populations is through hyphal anastomosis. *Verticillium dahliae* isolates able to anastomose their hyphae and form a stable heterokaryon are compatible, which led to their traditional classification into vegetative compatibility groups (VCG) [26]. The clonal structure of *V. dahliae* populations was first established by using VCG analysis, which was well supported later on by implementing different molecular marker approaches (see, for instance, López-Escudero and Mercado-Blanco, 2011 [16], and references therein). The current clonal population structure of *V. dahliae* is probably a dual consequence of a selection process due to the adaptation to crops and the clonal expansion of fit genotypes [27,28]. Single nucleotide polymorphisms (SNP) have been used in recent years for the genotyping of *V. dahliae* isolates to assign them to clonal lineages and determine the populations’ genetic structure [29]. Thus, nine distinct clonal lineages were identified by SNP analysis and shown to have originally arisen by recombination [30]. The four main VCGs identified in *V. dahliae* are VCG1, VCG2, VCG4, and VCG6, the first three being further divided into subgroups, A and B, based on the frequency, speed, and vigor of complementation [28]. In addition, supported by molecular genetic markers, the VCG2B lineage has been subdivided into three genetically distinct lineages, 2B^334^, 2B^824^ [30,31], and 2B^R1^ [30]. Moreover, population genomic analyses of *V. dahliae* revealed that clonal lineages historically arose by recombination, particularly lineages 2B^334^, 2B^R1^, and 6 [30]. Nevertheless, the assumption that isolates within a given VCG comprise genetically related isolates originating from a common ancestor has been questioned [31]. Phylogenetic analyses of individual and combined datasets indicated that for some *V. dahliae* VCG, this assumption is not necessarily true. Indeed, VCG may comprise a genetically heterogeneous group of isolates that are phylogenetically distant. VCG subgroups 1A and 1B are closely related and share a common ancestor [31]. However, isolates from VCG2A and 4B structurally and phylogenetically grouped together and distinctly from their “sister” VCG subgroups 2B and 4A, respectively [31,32]. In contrast to its clonal population structure, genes involved in meiosis were recently identified in the *V. dahliae* genome. This evidence suggested that *V. dahliae* has reproduced sexually in the past and, more interestingly, may still retain this potential [30,33].

It is well known that the severity of VWO attacks depends on the virulence of isolates that infect the tree. Focusing on *V. dahliae* isolates infecting olive, two pathotypes differing in their virulence level are traditionally described which correlate with specific VCG and clonal lineages: the defoliating (D) and the non-defoliating (ND) pathotypes. This classification is based on their ability to cause the severe defoliation of the tree (D pathotype) or a moderate wilting syndrome (ND pathotype) [16,34]. A second type of pathogenic variation in *V. dahliae* is based on the presence of two pathogenic races. Race 1 is defined by the presence of the effector gene *Ave1*, which confers avirulence to cultivars of tomato that carry the resistance gene *Ve1*. This gene encodes pattern-recognition receptors that recognize products encoded by *Ave1*, leading to a defense response against infection by race 1. In contrast, race 2 strains evade recognition due to the loss of *Ave1* and are able to infect Ve1 host plants [27,35]. The latest advances in understanding the relationships among pathotypes, races and lineages of *V. dahliae* reveal a degree of complexity. Isolates of race 1 belong to the lineage 2A and ND pathotype. The finding of race 1 in a single clonal lineage with identical *Ave1* sequences is consistent with the hypothesis that race 1 arose once in *V. dahliae*. Under this scenario, the hypothesis suggests that *V. dahliae* acquired *Ave1* from plants by horizontal gene transfer [35]. Molecular markers and virulence assays confirmed the well-established fact that the D pathotype is found only in lineage 1A, and all isolates in lineage 1A have the D pathotype [27]. Nevertheless, race 2 comprises seven lineages (1A, 1B, 2B^334^, 2B^824^, 2B^R1^, 4A, and 4B) and both pathotypes (D and ND); consequently, their understanding remains more complex [27]. Undoubtedly, a more comprehensive knowledge of relationships among the races, pathotypes, and clonal lineages will be of great help in VWO resistance breeding programs, aiding to identify and/or generate olive genotypes able to better cope with infections caused by more virulent isolates. 

## 4. Understanding the Molecular Bases of the *Verticillium dahliae*-Olive Interaction

Our knowledge of the biology and genetics of *V. dahliae*, as well as of the molecular bases of its interaction with different hosts, has hugely advanced during recent years. Major breakthroughs in *V. dahliae* research are due to the development and implementation of Next Generation Sequencing (NGS) approaches (for a review, see, for instance, [36]). Thus, the availability of several *Verticillium* spp. genomes [37,38] and the use of comparative genomics [39,40,41] and whole transcriptome analyses [42,43], coupled with powerful molecular and microscopy methodologies [44], has enabled the identification of an increasing number of genes involved in different aspects of *V. dahliae* pathogenicity and virulence [45]. The number of published studies in this regard is continuously increasing, and their comprehensive overview is beyond the aim of this review. We will now present a summary of some of the most relevant aspects. While some of the examples mentioned here refer to different *V. dahliae*–host interactions, the information generated serves well for our review purpose and can be extrapolated to the olive–*V. dahliae* pathosystem. 

### 4.1. Microsclerotia: The Main Infective Propagule under Natural Conditions

The biological cycle of *V. dahliae* when interacting with tree hosts, including the parasitic phase, has been described earlier [46]. In the particular case of olive, detailed microscopy imagery of the infection and plant tissue colonization processes is available [47]. It is well known that the natural infective propagules of *V. dahliae* (microsclerotia, MS) can endure in soil or plant debris for prolonged periods of time. This dormant structures can be spread by rain, irrigation water, human and animal activities, and agricultural tools and machines, distributing the pathogen to distant areas from the original inoculum source [48]. The infection process, and hence the parasitic phase, begins when MS germinate upon stimulation by host root exudates [26]. The identification of genes involved in MS production is thus of great relevance because of the epidemiological and pathogenic importance of these structures (i.e., central roles in pathogen survival and early steps in the root infection process). Yet, our knowledge on the genetics of MS production is limited. Since the focus of this review is VWO, we will just refer to some representative studies, avoiding an exhaustive description of the abundant literature recently generated on genes involved in MS biogenesis, as well as the relation between MS and virulence and the importance of the melanization process in MS generation. Interested readers are kindly invited to consult the available contributions on these issues (e.g., Luo et al. 2014; Luo et al. 2016 [45,49]).

Duressa and co-workers [50] identified more than 200 differentially expressed genes (DEG) involved in MS biogenesis, including pigment synthesis and secondary metabolism, cell growth, morphogenesis and cell death-related genes, carbohydrate-active enzymes and transport proteins. It is worth mentioning that nearly 50% of the identified DEG corresponded to hypothetical protein-coding genes, stressing the need to investigate many functions and processes yet to be uncovered. It has been demonstrated that VdHog1 and VdUDG mutants exhibited the reduced production and delayed maturation of MS [51]. The disruption of the *VdUDG* gene also inhibited spore formation [52]. The APSES proteins form a conserved class of transcription factors (TF) that control different aspects of the disease cycle, regulating the morphogenesis and other cellular processes in pathogenic species. In a recent study, the deletion of the APSES family TF Vst1 directly affected the development of *V. dahliae* MS as well as melanization and sporulation processes [53]. Similarly, the deletion of two nuclear TF, VTA3 and SOM1, led to MS alteration. The VTA3 deletion strain produced less MS than the wild type, and the SOM1 deletion strain was unable to form MS [54]. The involvement of additional TF such as basic leucine zipper (bZIP) TF has been investigated as well. For instance, the expression of one bZIP (VDAG_08640) that significantly increased during MS development [55] showed as down-regulated in the corresponding deletion mutant (VdMcm1) [56]. VDAG_08640 was also differentially up-regulated in a MS-forming strain of *V. dahliae* compared with a strain impaired in MS production [50]. While bZIP TF VDAG_08640 and VDAG_08676 were differentially expressed during MS development, the deletion of either gene did not affect MS formation. However, altered phenotypes such as the reduction in conidia production and virulence were observed in a VDAG_08676 mutant but not in a VDAG_08640 mutant [57]. 

It is well established that the development of fully functional MS in *V. dahliae* is linked to the biosynthesis of fungal dihydroxynaphthalene (DHN) melanin during the MS maturation process [58]. An impairment in synthesizing melanin implies a lack of MS production [50]. Besides this, there is a correlation between reduced MS and pigment production and the low survival of *V. dahliae* [59]. Therefore, the study of genes involved in these two highly connected processes can shed light on pathogenicity and virulence. Nevertheless, the relationship between melanization and *V. dahliae* virulence seems sometimes contradictory. Indeed, melanin-deficient mutants commonly show reduced virulence, also correlated to their inability to produce MS [49,59], although this rule does not always apply [60]. 

Finally, the role of autophagy in soil-borne fungal biology and physiology has also been studied, although its involvement in these processes is still poorly understood. Of particular interest is how autophagy processes have evolved in plants and phytopathogens in relation to defense and infection strategies, respectively [61]. The results obtained with two autophagy-associated genes are worth mentioning. Indeed, while phenotypes of *V. dahliae* mutants lacking either *VdATG8* or *VdATG12* genes (related to autophagy) showed similar MS to those generated by the wild-type strain, they showed an altered aerial hyphae development and reduced conidiation [62].

### 4.2. Understanding the Olive–Verticillium dahliae Interaction

Soil-borne pathogens have developed different strategies to successfully invade the host plant [63]. Regarding to *V. dahliae*, a number of genes involved in fungal adhesion or root penetration have been described in recent years [54,64]. Interestingly enough, some of these genes are also involved in MS production (see above), stressing the fact that the production of these resting structures, pathogenicity, virulence, and host infection are processes intimately interconnected [36]. Our knowledge on the physiological, biochemical, and genetic mechanisms underlying the olive–*V. dahliae* interaction has increased during the last decade. Indeed, different aspects related to pathogenicity and virulence as well as host tolerance/resistance to VWO were recently unveiled.

**Figure 4:**
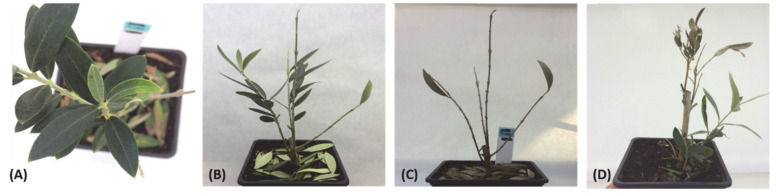
Symptoms observed upon the artificial inoculation of the defoliating pathotype of *Verticillium dahliae* in olive plants cultivar Picual. (**A**) Chlorotic and distorted leaf; (**B**) partial severe defoliation (affecting some stems) of green leaves; (**C**) fully defoliated dead plant; (**D**) olive plants may undergo the so-called natural recovery phenomenon (see, for instance, López-Escudero and Mercado-Blanco, 2011 [16]).

To successfully invade the host, the pathogen must first overcome a physical defense barrier composed of lignin, a major component of the plant cell wall, and suppress the activity of secondary metabolites and antimicrobial compounds released by the host as part of the defense response deployed against the invader. Therefore, the production of cell wall-degrading enzymes is one of the pathogenicity factors contributing to Verticillium wilts [65]. Once the pathogen is able to surmount the mechanical defense layer of the roots, it invades the xylem vessels, impairs water transport, and causes the typical wilt syndrome, with symptoms such as early senescence, chlorosis, necrosis, stunting, defoliation, and, in some cases, the death of the plant (Figure 4) [26,34,66,67]. 

The loss of hydraulic conductivity is attributed to vessel occlusion by tyloses and gels produced by the plant as a response to the infection by the pathogen aiming to halt its spread [66,68]. Cavitation induced by vascular pathogens also reduces water conductivity in the xylem due to the air filling of the vessels [69]. Starch hydrolysis is considered a mechanism to restore the hydraulic conductivity after cavitation. The degradation of starch in the parenchyma cells of the xylem produces soluble sugars that are released into the vessels, thereby promoting an osmotic flux of water into their lumen [67,70]. The correlation between the starch content and density of occluded vessels has been recently studied in olive cultivars differing in susceptibility to VWO [67]. The results showed that the infected plants of the susceptible cultivars Arbequina and Picual displaying moderate to high disease severity levels, presenting an increase in the density of occluded vessels. In contrast, the starch content decreased in these cultivars. Interestingly, in the resistant cultivar Frantoio, the starch content and density of the occluded vessels in the stem did not differ from the control plants. This may be explained by a quick defense response and the activation of physical and chemical mechanisms in the root and basal stem of this cultivar [67,71] that restricted the shoot colonization and, consequently, the effect of vessel cavitation and occlusion [67]. In this regard, upon pathogen attacks plants have developed a set of defense mechanisms such as the induction of the antioxidant system, activation of defense signaling pathways by production of reactive oxygen species (ROS), and reinforcement of the cell wall by the deposition of lignin and suberin at the site of infection [72]. While ROS are molecules highly toxic for the plant cells that increase under stress situations, they also act as signaling molecules involved in pathogen defense responses [72,73]. After *V. dahliae* inoculation, a significant H_2_O_2_ burst was observed in olive plants, especially in resistant cultivars [72]. During the first stage of the infection process, resistant and moderately susceptible olive cultivars maintained high levels of H_2_O_2_, while susceptible cultivars showed a significantly lower content [72,73]. To dissipate the toxic effects of ROS, plants have developed a set of highly regulated enzymatic and non-enzymatic mechanisms [72,73,74]. For instance, the observed significant increase in superoxide dismutase activity in the resistant cultivar Sayali evidenced the importance of this enzyme in the antioxidative defense mechanism of the olive plant against *V. dahliae* infection. In addition, a suppression of catalase activity was reported in the pathogen-inoculated resistant cultivar two days after inoculation (DAI), which could explain why this cultivar accumulates much more H_2_O_2_. By contrast, the significant up regulation of catalase and ascorbate peroxidase activities from four DAI in the inoculated plants of the susceptible cultivar Chemlali may explain why this cultivar did not display an ROS burst [72,73]. 

Chitin is the main component of the pathogen cell wall. Thus, cell wall-degrading enzymes such as chitinases and β-1,3-glucanase are generally involved in the plant defense against fungal pathogens. Indeed, chitinase and β-1,3-glucanase activities were induced earlier in the resistant cultivar Sayali than in susceptible “Chemlali” plants. The early up-regulation of genes coding for both enzymes allowed the host to anticipate the spread of the pathogen, restricting its growth at the site of infection [72]. Finally, polyphenols, soluble sugars, and lignin content were positively correlated with the resistance level of the cultivars. The higher concentrations of these compounds in resistant cultivars suggest their implication as key factors in olive defense against *V. dahliae* [68,73,74].

The activation of plant defense pathways is also mediated by the plant hormones salicylic acid (SA), jasmonic acid (JA), and ethylene (ET), which are well known to play central roles in the defense against pathogens. The up-regulation of SA-related genes was simultaneously accompanied by the H_2_O_2_ burst in the resistant cutivar Sayali. Meanwhile, the analysis of the expression patterns of *JZIM* (jasmonate ZIM domain) and *bHLH* (BHLH binding factor responsive to JA) revealed that both genes were strongly induced in “Sayali” but not in “Chemlali” (susceptible). Likewise, an ET response factor (*ERF*) was strongly induced at the early stage of infection in the resistant cultivar but not in the susceptible one, suggesting that ET may play a role in the enhancement of olive resistance to *V. dahliae* [72].

Nevertheless, an in-depth knowledge of the mechanisms underlying the response of olive plants to *V. dahliae* attack, as well as those related to resistance to VWO, requires further molecular and physiological analyses, providing a more holistic perspective. The availability of powerful NGS-based methodologies has undoubtedly helped in this endeavor. Earlier, and by using Suppression Subtractive Hybridization (SSH), many genes differentially expressed during the interaction of the D pathotype with the tolerant cultivar Frantoio were identified [71]. The expression pattern of some of them was then evaluated over time upon inoculation with *V. dahliae* in other cultivars differing in susceptibility to VWO. For instance, the expression pattern of *GRAS1*, a TF involved in plant response, was down-regulated in tolerant cultivars. On the contrary, “Picual” plants (VWO susceptible) showed a sharp decrease in *GRAS1* expression levels over time. Similarly, the expression of *DRR2* (coding for a disease resistance response protein) was down-regulated in tolerant cultivars but up-regulated in “Picual” plants [71]. Thus, the differential expression patterns of these two genes were proposed to be used as markers of the tolerance level of olive cultivars to *V. dahliae*.

Later on, and by using RNAseq, the co-transcriptomes of *V. dahliae* (D pathotype) and olive (cv. Picual) were generated from a time-course experiment aiming to determine the responses of both the host and the pathogen during the early moments of the interaction [43]. Up to date, this is one of the few examples so far available of a co-transcriptome study, offering the unique opportunity to analyze the genetic dialogue established between a pathogen and its host at the whole transcriptome level. A newly assembled olive transcriptome was further generated upon enrichment with the transcriptome generated during the interaction of “Frantoio” plants with the same D isolate, eventually generating the so-called “PicFra” transcriptome. The main conclusion of these studies was that VWO tolerance displayed by “Frantoio” plants is a consequence of a complex and multifaceted process in which both the basal and early *V. dahliae*-induced differential transcriptomic responses are involved [75]. Moreover, the transcriptomic response of the pathogen was also different depending on the cultivar with which it interacts. Indeed, the comparative quantification of *V. dahliae* mRNA amounts indicated that the biomass of the pathogen was significantly higher in the roots of “Picual” than in those of “Frantoio”. By extension, a very high number of *V. dahliae* unigenes coding for niche-adaptation, pathogenicity, virulence, and MS development were induced in “Picual” plants [42]. Analyzing transcriptomic data from the pathogen and the host, it can be concluded that VWO susceptibility can be largely explained by the absence of basal and some pathogen-induced transcriptomic responses in susceptible varieties, thereby favoring the proliferation of the pathogen, enhanced transcriptional activity, colonization, and further dissemination in host tissues [42]. These results are in agreement with those from other studies in which the biomass of the pathogen was also more abundant in the roots and stems of VWO-susceptible olive cultivars than in resistant ones [68,73]. A somehow similar scenario has been reported in resistant wild olive compared with “Picual” plants. In this case, the mean amount of *V. dahliae* D pathotype DNA in the stem tissues of this susceptible cultivar was >90 times higher than the highest mean value scored in the resistant wild olive clones “Ac-13”, “Ac-18”, and in cultivar Frantoio [76].

The lineage-specific (LS) regions are genome sequences, unique or shared among a subset of strains, that contain hundreds of genes [77]. Moreover, these LS regions are enriched in in planta-induced putative effector genes and transposable elements [39,78]. Genomic studies have provided evidence supporting that these regions significantly contribute to virulence and niche adaptation [39,78,79]. Recently, the deletion of seven genes, designated as *VdDf* genes and encoded in an LS region, produced the non-defoliating phenotype on cotton, olive, and okra [80]. The complementation of two of them restored the D phenotype. Thus, this study enabled the association of strain-specific LS regions (LSRs), called G-LSR2, to the D pathotype of *V. dahliae*. Furthermore, the gen *VdDf7* shared homology with proteins involved in the biosynthesis of N-lauroylethanolamine (N-acylethanolamine (NAE) 12:0), a compound that induces defoliation [80]. 

Finally, the application of crude extracts containing *Verticillium* toxins has been demonstrated to elicit plant defense responses in olive. Indeed, crude extracts from representatives of the D and ND pathotypes induced the curling of leaves and browning of olive twigs in tolerant (Frantoio) and susceptible (Leccino) cultivars. Defoliation eventually was observed in both cultivars. Overall, the symptoms were similar to those observed when both cultivars were naturally infected by *V. dahliae* [81,82]. In addition, the phytotoxic metabolites present in the crude extracts were able to induce physiological changes affecting the transpiration stream, cell membrane integrity, and chemical defenses associated with plant secondary metabolites involved in stress resistance/tolerance [82].

## 5. Advances in *Verticillium dahliae* Detection 

VWO is widely distributed worldwide and its devastating effects are well known. Disease management becomes difficult due to the presence of MS that remain dormant for long time in the soil. In addition, infected but asymptomatic plants favor, among other epidemiological factors, the silent spread of the disease. New detection and diagnosis technologies have been developed and implemented, complementing and improving approaches already available and mostly based on different PCR-based procedures [16,34]. These are essential for the early detection of VWO, even when symptoms are not yet visible, as the first crucial step in the integrated disease management strategy.

### 5.1. Detecting the Pathogen: From Remote Sensing

The visual inspection of VWO symptoms in the field, particularly at early stages of the disease development, may constitute an expensive and time-consuming endeavor. This is due to the usually large size of olive orchards in some regions and the unfeasibility of performing disease diagnosis tests for each tree. Currently, the use of remote sensing constitutes a revolutionary technology employed in assessing crop status under field conditions, enabling the detection of the pathogen even at early stages of disease development. Remote sensing is a set of advanced technologies based “on the information obtained from an object without physical contact, by measuring the electromagnetic energy reflected/backscattered or emitted by the surface of the target object” [83]. The spectral measurements acquired by portable instruments, called proximal sensing, are also included in this definition [83]. These methods are rapid, non-destructive, and cost-effective, enabling the user to collect data rapidly compared to the usually time-consuming diagnosis/detection by ground-based techniques [84,85]. As mentioned above, *V. dahliae* infects the plant through the roots and colonizes its vascular system, blocking the water flow to the aboveground organs and eventually leading to the characteristic wilting syndrome [16]. This effect translates to changes in the spectral reflectance in the aerial organs of the plant, changes that can be measured. For instance, a decrease in the transpiration rate due to the occlusion of xylem vessels induces stomata closure, thereby reducing evaporative cooling and increasing the canopy temperature. In addition, the reduction in photosynthesis caused by *V. dahliae* infection leads to an increase in the dissipation of energy by fluorescence [85]. The use of thermal, multispectral, and hyperspectral imagery acquired with unmanned aerial vehicles (UAV) represents a useful approach to detect VWO at early stages of disease development. Information provided by these indexes goes a step further and can be used as indicators to detect and differentiate the presence of moderate to severe damage caused by *V. dahliae* attacks [86]. Therefore, the data obtained by UAV showed that normalized olive canopy temperature, chlorophyll fluorescence, and blue/blue–green/blue–red ratios (B/BG/BR indices) were found to be the best indicators of early stage infection by the pathogen, while the Photochemical Reflectance Index (PRI), structural, chlorophyll, and carotenoid indices detected only moderate to severe *V. dahliae* infections [85,86]. Furthermore, a very recent study even included the use of indices derived from RGB (red-green-blue) images for the first time to assess VWO in combination with control strategies, such as the use of organic amendments [84].

Despite the advantages discussed here, the main challenge these techniques must face is the accurate differentiation between biotic and abiotic stresses leading to the same effects. *Verticillium dahliae* infection directly affects the physiological status of the olive tree, causing effects that can also be produced by abiotic stress (i.e., drought), or even by other vascular or root-damaging pathogens. Notwithstanding these current limitations, remote sensing is predicted to be the principal methodological approach for data acquisition from agricultural fields, assisting in the early diagnosis of diseases such as VWO.

### 5.2. To on Ground Diagnosis

Even though remote sensing techniques are becoming (and will definitively be) more relevant, the traditional molecular methods for detecting the target pathogen, both in planta and in soil, remain the most frequently used diagnosis tools. The latter are more accessible to farmers and agricultural extensionists, and usually allow the unequivocal detection of the pathogen. As mentioned above, the pathogen infection may cause similar symptoms to those produced by other abiotic/biotic stresses. In addition, many plants can be infected but remain asymptomatic [87,88], or may recover from symptoms later on [89], hindering their detection and promoting the spread of the disease. Advances in the diagnosis and detection of VWO have been mostly focused on the improvement of the detection threshold of the pathogen and on the refinement (i.e., accuracy and effectiveness) of the available procedures [90]. There is a study regarding in planta detection and, to the best of our knowledge, advances relating to the improvement, evaluation, and comparison of available PCR-based approaches [87]. This exhaustive and comparative study concluded that some of the published real-time qPCR protocols were not truly specific for *V. dahliae*, or were ineffective when used in a plant tissue extracts background. The available literature about *V. dahliae* detection in soil has been moderately enriched during the last few years. Yet, traditional identification based on PCR procedures is the most frequent methodological approach. This relies on the isolation and purification of DNA collected from soil samples that is then subjected to PCR protocols using specific primers. Alternatively, *V. dahliae* can be grown from soil samples using appropriate culturing media and then morphologically and molecularly identified [16]. To overcome some of the limitations of these methods, mainly aiming at reducing the costs for insufficiently equipped labs, a loop-mediated isothermal amplification (LAMP)-based procedure was developed with satisfactory results [91]. Besides being more cost effective (no need of expensive equipment), the DNA purification step is skipped. The LAMP technique is growing in popularity for the detection of many human, animal, and plant pathogens [92]. However, the available information on its use in *V. dahliae* detection is still limited. Due to the need to detect the least amount of MS per soil sample, a method that combines the conventional wet-sieving [93] with SYBR Green I-based real-time qPCR, namely the wet-sieving qPCR method [94], has revealed as an useful tool to quantify *V. dahliae* in soil samples. This method can consistently quantify *V. dahliae* propagules at as low as 0.5 MS/g in soil, which is sensitive enough for most research studies and practical applications. Compared with other techniques, this is also the lowest detection limit among assays currently available for *V. dahliae*, as the addition of a TaqMan probe has reported a detection limit of 1-2 MS/g in soil [95].

Finally, a peculiar way to detect *V. dahliae* has been investigated. Microorganisms are able to produce a broad range of metabolites, including volatile organic compounds (VOC). These compounds are mostly undetectable by humans but some animals, among them domestic dogs, are able to detect these substances present even at very low concentrations. Remarkably, one study has explored the possibility of detecting *V. dahliae* by exploiting the highly sensitive olfactory sense of dogs. A specifically trained canine unit was thus able to detect the pathogen with a 97% success rate and 95% specificity under a controlled working environment. A preliminary assay with real infected trees showed excellent effectiveness, with the dogs providing positive responses for 19 out of 20 olive trees affected by VWO [96]. To our knowledge, this is the first study reporting the detection of *V. dahliae* by the emission of specific VOC. Despite the novelty of this methodology, it is undeniable that more studies will be necessary for its implementation. Moreover, since VOC production can be affected by plants, microbes, environmental conditions, etc., this methodology seems to be difficult to standardize. In addition, to corroborate the presence of the pathogen, detection by molecular methods will be necessary.

## 6. Advances in the Management of Verticillium Wilt of Olive

The effective control of Verticillium wilt is very difficult for reasons comprehensively reviewed elsewhere [16,18,19]. The true fact is that recent surveys and reports continuously alert about the spread of the disease to new areas or about the increase in its incidence and severity in regions where the pathogen is present, particularly due to the growing prevalence of the highly virulent D pathotype [97]. Therefore, the well-known epidemiological factors contributing to its expansion (the diversity of efficient dispersal method, the use of infected planting material, inadequate agronomical practices, etc.) are still in effect, despite all efforts made by scientists, extension personnel, and diligent farmers to handle the disease and hamper the dispersion of the pathogen. In addition, the endurance of MS in soil, the long lifetime of trees permanently exposed to *V. dahliae*-infective propagules, the broad range of hosts, the absence of methods to cure infected trees, and the presence of pathotypes (D and ND) displaying differential virulence explain the difficulty of managing the disease. The implementation of an integrated disease management framework is thus the only way to either successfully “live with the problem” (in areas where the pathogen is present) or to avoid its spreading to new areas where olive is being cultivated. This holistic approach, combining both preventive (pre-planting) and palliative (post-planting) measures, is the best strategy to confront the disease and mitigate pathogen dispersal [16]. We now offer an overview on recent advances in VWO control measures. 

### 6.1. The Continuous Search for Sources of VWO Tolerance/Resistance 

The development of VWO tolerant/resistant genotypes has become a major objective for olive breeding programs [98]. The use of resistant cultivars is considered as the most economic, environmentally friendly, and efficient control measure for the disease, and efforts to search for new resistant genotypes and/or evaluate available cultivars were previously reviewed [16,19]. Unfortunately, most of the olive cultivars so far evaluated are susceptible or extremely susceptible to the pathogen, particularly those that are broadly cultivated in olive growing areas (e.g., “Arbequina” and “Picual”) [99,100]. During the last 15 years, many olive cultivars with interesting agronomical and commercial characteristics have been assessed as to their VWO resistance level [100]. In this sense, the World Olive Germplasm Bank located in Córdoba (Spain), as well as other germplasm collections elsewhere, represents an excellent source of cultivars and wild genotypes to be screened for disease tolerance/resistance [101,102]. 

One of the main problems the studies evaluating new cultivars face is the difficulty to replicate both the pathogen infection process and the disease development under (usually variable) natural conditions. Moreover, to find a correspondence between the results obtained under controlled and field conditions constitutes a true challenge. The amount of *V. dahliae* inoculum applied or present in infested soils [100,103], different inoculation [102,104] and inocula production [105] methods, or different temperatures [106] are variables that may influence the onset, incidence, severity, and virulence of the disease. When performing olive genotype evaluations under field conditions, the scenario becomes even more complex, since assays can be largely influenced by highly variable environmental, climatic, and pedological factors as well as different crop management practices [24]. While widely used cultivars are unfortunately susceptible to VWO (see above), others such as “Empeltre”, “Koroneiki”, “Changlot Real”, and “Frantoio” have demonstrated high levels of tolerance to the D pathotype of *V. dahliae* under controlled [98,107] and field [100] conditions. Nonetheless, in this latter scenario, VWO symptoms were detected in some of the plants [100]. Besides this, the cultivars Escarabajillo, Menya, and Sevillana de Abla have been shown to display high levels of resistance to the D pathotype as well, performing even better than “Frantoio” under greenhouse conditions [102]. Results from field experiments are still needed to confirm the level of resistance to VWO in these local cultivars.

Some studies have focused on the identification of potential new sources of resistant cultivars/genotypes which are only grown in very specific geographical areas [76,108,109]. For example, the Greek cultivar Kalamon was classified as resistant (greenhouse experiment) to the D pathotype [110]. In contrast, the Iranian indigenous cultivars Rowghani, Marry, and Zand were classified as susceptible under the same experimental conditions. In Spain, the cultivars Cornezuelo de Jaén, Verdial de Badajoz, Jaropo, Negrillo de Estepa, Jabaluna, Ocal de Alburquerque, Asnal, and Racimal were reported as resistant or tolerant under greenhouse conditions, all of them showing just minor disease symptoms [111]. These results are promising, although it must be taken into account that, in addition of being resistant to the disease, desirable agronomic traits such as short juvenile period, early bearing, industrial suitability, high oil content, and the diversity of olive oil composition must also be present/preserved in the new selected varieties [112,113]. However, these resistant cultivars are rather local and will be unlikely used in commercial olive production. Indeed, the choice of a given cultivar for establishing new olive orchards is mainly driven by edaphic and climatic conditions, certificates of geographical origin for oil production, and market requirements [76]. By implementing crossbreeding techniques, new cultivars or well-established cultivars with modified/improved phenotypic traits can be developed. For instance, “Sikitita”, the result of a cross between “Picual” (susceptible to VWO) and “Arbequina” (moderately susceptible to VWO) was the first cultivar adapted to high-density hedgerow orchards [112]. Unfortunately, this variety has been reported as moderately susceptible to VWO [98]. Therefore, its use in old or newly established orchards should be limited to soils where the presence of *V. dahliae* is discarded.

Regarding breeding for VWO resistance, different studies have confirmed that the cultivar Frantoio confers a high level of resistance to *V. dahliae* to its progeny under artificial conditions [108]. However, not all resistant cultivars conferred resistance to their offspring. For instance, the cultivars Changlot Real and Empeltre, qualified as resistant to *V. dahliae* [98,100,107], mostly generated susceptible offspring in greenhouse experiments when they were used as genitors, even from crosses among them, presumably due to incompatibility phenomena [98]. 

Wild olive germplasm constitutes an interesting and valuable source of resistant genotypes to the D pathotype of *V. dahliae* [76,114]. Fifty-six genotypes, including wild olives from the related subspecies *O. europaea* subsp. *guanchica* (indigenous from Canary Islands) and genotypes originating from crosses between “Picual” and wild olive trees were evaluated under controlled conditions upon inoculation with a representative isolate of the D pathotype. Thirteen genotypes, two of them belonging to subsp. *guanchica* populations, three genotypes from one of the “Picual” x wild olive crosses, and eight wild olives from different locations were classified as resistant [114]. In this way, the wild olive genotype and its progenies, even from crosses with the susceptible “Picual”, represent a source of resistance to VWO. Moreover, this was the first report of resistant *guanchica* genotypes. In view of these promising results, the use of wild olive genotypes opens new avenues in the search of sources of resistance to *V. dahliae*. Nevertheless, it is worth mentioning that the main goal of these studies is the identification of highly resistant rootstocks adapted to *V. dahliae*-infested soils and capable of satisfying farmers’ and consumers’ demands for high yield and good oil quality. Currently, none of the resistant wild olives are being used in commercial fields. The validation of these cultivars as resistant to other olive diseases could increase their interest. For instance, the VWO-resistant wild olive clones “Ac-13” and “Ac-18” [76] were evaluated in co-inoculation experiments (*V. dahliae* and the phytopathogenic nematode *Meloidogyne javanica*). Unfortunately, the experiments did not yield promising results. Both of the clones were susceptible to *M. javanica*, although they retained their resistance to *V. dahliae* [115]. This fact validates the claim that both clones could be excellent rootstocks and of paramount importance for the production of agronomically adapted and commercially desirable olive cultivars. Related to this, Vertirés^®^ [27,76], a trademark of different types of grafted olives over clones “Ac-13” and “Ac-18” that is highly resistant to all the pathotypes and races of *V. dahliae* was developed. In addition, the roots of Vertirés^®^ are treated with the biocontrol fungus *Trichoderma asperellum*, which also protects against the soil-borne oomycete *Phytophthora* spp. and the mycorrhizal fungus *Rhizophagus irregularis* (formerly *Glomus intraradices*) in order to reinforce the root development and better tolerate water stress [116]. 

Finally, genetic transformation can be a useful approach for the development of resistance against several plant pathogens. *Aspergillus giganteus* is known to produce one protein with antimicrobial activity called antifungal protein (AFP). This protein interrupts the normal behavior of the plasma membrane, and may enter the host cell and promote the neutralization and condensation of DNA [117]. The xpression of the *afp* gene in transgenic plants effectively controlled pathogens such as *Fusarium graminearum* in wheat [118], *Rosellinia necatrix* in olive [119], and *Magnaporthe grisea* in rice [120]. Nevertheless, the constitutive expression of the *afp* gene in olive did not protect against VWO [119]. In a recent study, transgenic olive plants expressing the *NPR1* gene from *Arabidopsis thaliana* were generated to evaluate their differential response to *V. dahliae*. The *NPR1* gene is a key regulator in the systemic acquired resistance (SAR) pathway. Regrettably, the heterologous expression of the *NPR1* gene in transgenic olives did not confer resistance to the D pathotype of *V. dahliae*, although it improved the plant response to the ND pathotype [121].

### 6.2. The Key Is in the Water Treatment

As discussed above, irrigation water is a demonstrated dispersion source of *V. dahliae* infective propagules, contributing to increases VWO disease incidence and severity in some areas. Therefore, avoiding the spread of *V. dahliae* by irrigation systems must be a key measure within integrated disease management frameworks [48]. The use of sand filters failed to prevent the spread of *V. dahliae* through the drip-irrigation systems of olive orchards [122]. Consequently, efforts to implement feasible water disinfection strategies have been conducted during recent years. Adding commercial disinfectants to irrigation water has revealed an excellent measure to control and reduce the viability of *V. dahliae* propagules, reaching in some cases up to 100% effectiveness [123,124,125]. 

Hydrogen peroxide is classified as an environment-friendly disinfectant, with a broad spectrum of activity and absence of persistent toxic by-products [126]. OX-VIRIN^®^ and OX-AGUA AL25^®^ are two commercial water disinfectants based on hydrogen peroxide in combination with other oxidizing and non-oxidizing agents, respectively. In addition to the advantages mentioned above, they can be applied in a wide range of circumstances. Indeed, the application of OX-VIRIN^®^ and OX-AGUA AL25^®^ produced a decrease in MS viability both under in vitro conditions [123,124] and in *V. dahliae* naturally [123] or artificially infested [127] soils. Likewise, the effectiveness of the treatments was verified in soil with plants by evaluating the VWO development. Thus, “Picual” and “Arbequina” plants inoculated with *V. dahliae* and subjected to these disinfectants showed a lower disease incidence compared with the untreated ones in experiments conducted in controlled growth chambers [125]. Under field conditions, olive trees transplanted into a soil previously treated with the disinfectants effectively withstood the disease [128]. Similarly, a reduction in symptoms and disease incidence was found in olive trees transplanted into an artificially infested soil subjected to disinfestation with respect to untreated water control [127]. Moreover, the addition of these disinfectants neither affected the growth parameters (shoot weight and foliar area) nor caused phytotoxicity [125,127] and, surprisingly, improved the growth of the trees [127]

In conclusion, water treatment with disinfectants may constitute a very effective approach to control the dispersion of *V. dahliae* propagules within an integrated VWO management strategy. On the one hand, it can considerably reduce the inoculum level of *V. dahliae* in irrigation water (suppressive measure). On the other hand, it can prevent both the introduction of pathogen propagules in *V. dahliae*-free areas and the increase in the inoculum density in already infested soils (preventive, exclusion measure).

### 6.3. Heat Treatments in Sanitation of Olive Plants

The knowledge of the most favorable soil temperature range for the onset and development of VWO is instrumental to developing new approaches to manage the disease. It is well known that MS are the most thermotolerant structures of *V. dahliae* [129]. In addition, MS constitute one of the best sources to spread the disease. Therefore, any attempt to reduce the number of viable MS constitutes a key element to control Verticillium wilts, including VWO. It has been demonstrated that MS are better produced at 20±5 °C under laboratory controlled conditions [130]. Experiments carried out in greenhouse conditions showed that soil temperature is critical for VWO development. Thus, infection by the D pathotype was promoted by soil temperatures in the 16-24 °C range in the cultivar Picual and in the 20-24 °C range in “Arbequina” plants. For infections caused by the ND pathotype, soil temperatures ranging from 16 to 20 °C were the most favorable ones [131]. In all cases, the disease incidence, disease severity, and extension of the stem vascular colonization decreased when the soil temperature increased up to 32 °C. The application of heat treatments has been studied to reduce or eliminate different pathogens in citrus species [132,133], grape [134,135], or pecan crops [136]. The evidence of VWO control by heat treatment was documented by Morello and workers [137]. In this study, *V. dahliae* was eliminated from infected 1-year-old olive plants after moist hot air treatments (42-44 °C for 6–12 h). This treatment is simply based on the reliable monitoring of temperature and is not time consuming. 

### 6.4. Organic Amendments: A Second Life for Agricultural Waste to Control VWO

Large amounts of agro-industrial products are produced during, for instance, the processing of olives, grapes, or cork. These sub-products (e.g., the semisolid residue from the extraction of olive oil by the two-phase system (i.e., solid olive oil waste), grape marc, cork waste, and lactic acid), while posing serious environmental problems may have advantages and the potential to improve soil health and help in the control of soil-borne diseases [138]. 

The particular case of sub-products from the olive oil production process constitutes a serious management problem due to their phytotoxicity and semisolid nature [139]. Consequently, the huge amount of this residue generated in olive-producing countries needs urgent economic, environmentally friendly and sustainable approaches for its proper management. Strategies to explore new uses of this organic waste are thus encouraged [140,141,142,143]. Indeed, several studies highlight the added value of these wastes as fertilizers when added to the soil due to their high organic matter and mineral contents. In addition, the suppressive effects of olive mill waste water against soil-borne plant pathogens (e.g., *Fusarium solani*, *Rhizoctonia solani, Sclerotinia sclerotiorum*, *V. dahliae*…) has also been studied [140,144].

Organic amendments (OM) include solid and liquid materials, or mixtures of them, with a highly diverse composition and from a wide range of animal and plant origins. Composting is usually required to reduce their phytotoxicity prior to their use. They are applied as natural fertilizers, contributing to reducing heavy inputs of synthetic agrochemicals, thereby minimizing residues originating from farming activity. Despite the numerous advantages of OM, their use in controlling VWO has not been frequently reported, although some examples are available [145,146]. Recently, the ability to reduce the mycelial growth and MS viability of *V. dahliae* by five OM from olive oil waste compost, cow manure, vermicompost and two commercial compost teas, and combinations among them, was evaluated [145]. Results showed that the application of OM, especially solid olive oil waste compost and compost tea, inhibited or reduced the viability of *V. dahliae* MS in soil as well as the disease incidence in olive plants. Similarly, the co-compost of olive mill waste and olive leaves delayed the onset of VWO symptoms in “Arbequina” plants compared with a standard substrate (coir fiber). Interestingly, grape marc compost and a co-compost of olive mill waste and olive leaves reduced the number of infective MS in the olive rhizosphere [147].

The effectiveness of OM is sometimes a consequence of synergies with other substances or microorganisms, resulting in much better results compared with those obtained when the OM is used alone [148]. For instance, the addition of lactic acid to the solid olive oil waste compost provided a consistent reduction in MS viability [145]. In other cases, the effectiveness of the OM is determined by the addition of a particular microorganism. In this way, the mixture of olive mill waste with *Bacillus amyloliquefaciens* and *Burkholderia cepacia* produced a much better control of VWO compared to that observed with the use a *T. asperellum* TV1-based biofungicide [146]. The application of these amendments at the nursery stage may pose interesting practical advantages, mostly from the economic and efficiency points of view. However, further studies under natural field conditions are essential to demonstrate their effectiveness against VWO [149].

The ability to suppress VWO by using OM relies on different mechanisms that are yet insufficiently understood. The inhibition of the pathogen’s growth may be attributed to biocontrol exerted by specific components of the microbial communities present in the OM and/or that are stimulated upon the addition of the OM to the soil, by means of antibiosis, competition, or parasitism [150,151]. The different physical-chemical properties of OM, such as variations of pH; EC (dS/m); the concentration of K^+^, Ca^+^, PPO_4_^3^, N-NO_3_^-^, and N-NH_4_^+^; enzymatic diversity; β-glucosidase activity; oxygen uptake rate; or phosphatase activity may influence the effectiveness of OM treatments and may be used as predictors of the suppressive capacity of these composts against *V. dahliae* [147,152,153]. 

### 6.5. The Continuous Search for Effective Biological Control Agents Against VWO and the Mechanisms Involved

Biological control represents an interesting, sustainable, and environmentally friendly approach within the integrated management strategies of VWO. Recently, Deketelaere and co-workers [154] have comprehensively and critically reviewed the use of biocontrol agents to confront Verticillium wilts in general. We now focus on the recent advances on this topic for the particular case of VWO. 

The genome of the well-known BCA against VWO *Pseudomonas fluorescens* PICF7 has been sequenced [155]. Regarding the mechanisms involved, a mutant analysis has revealed that neither siderophore (pyoverdine) production nor swimming motility are required for the biological control activity of strain PICF7 against *V. dahliae*. Moreover, these phenotypes are not involved in the ability of PICF7 to colonize the olive root interior either [156]. Interestingly enough, the strain PICF7 is also able to reduce Verticillium wilt symptoms in the model plant *A. thaliana* [157] and to promote plant growth in a distant plant species such as barley (*Hordeum vulgare* L.) [158]. In contrast, no growth promotion was observed in wheat (*Triticum aestivum* L.), even though the strain PICF7 displayed similar endophytic behavior in both cereals. However, no evidence of endophytic colonization by PICF7 was found in *A. thaliana*. Similarly to the results obtained for olive, siderophore production and swimming motility were not involved in the biocontrol of Verticillium wilt in this model plant. Finally, evidence of induced systemic resistance against *Botrytis cinerea* was reported in this study system [157]. This raised the question of whether systemic defense responses could be effective in distant tissues upon root colonization by the strain PICF7. Indeed, by using SSH it was demonstrated that PICF7 is able to trigger, among others, a broad range of defensive responses upon root colonization. This was not only true at the local level (i.e., roots, the original inoculation site) [159] but also in aerial tissues [160]. While the up-regulation of genes involved in responses to different stress was demonstrated, the systemic responses triggered by PICF7 seemed to be ineffective against another olive pathogen, *Pseudomonas savastanoi* pv. *savastanoi*, causing olive knot disease [161]. Later on, and by using a split-root system, effective biocontrol was not observed when *V. dahliae* and the strain PICF7 were spatially separated [162]. Overall, these results suggested that VWO biocontrol by PICF7 is likely a consequence of competition for niche/nutrients, antibiosis, and plant responses triggered by the BCA that act in a concerted way over time and space. 

The search for a novel BCA against VWO is ongoing and promising results have been gathered, although mostly under controlled experimental conditions. Recently, three new *Pseudomonas* spp. strains have been thoroughly characterized, including the genomic level [163,164]. The strains *Pseudomonas* sp. PIC25, *P. indica* PIC105, and *Pseudomonas* sp. PIC141 were isolated from healthy nursery-produced olive (cv. Picual) plants. The three strains showed in vitro antagonism against several olive pathogens, including the D pathotype of *V. dahliae*. While *P. indica* PIC105 (for the first time, a representative of this species was described as a BCA) was the most effective at antagonizing olive pathogens, in planta assays demonstrated that the strain PIC141 was the most effective against VWO under non-gnotobiotic conditions, with a comparable performance to that observed for the well-characterized BCA PICF7. This emphasizes the absolute need to perform in planta experiments to truly demonstrate the biocontrol effectiveness. In their study, Gómez-Lama Cabanás and co-workers [163] have proposed a flowchart of actions aiming to isolate, identify, and characterize novel and effective BCAs from the root/rhizosphere. Obviously, this scheme can be followed in other soil-borne pathogen–plant scenarios. Field experiments to verify the effectiveness of these BCA under natural (usually adverse and diverse) conditions are particularly encouraged within this scheme [163]. 

Bacterial BCA against VWO are not limited to *Pseudomonas* spp. members. For instance, following the same scheme of actions mentioned above, three *Bacillales* strains (PIC28, PIC73, and PIC167) from the olive rhizosphere were also identified as effective BCAs against the D pathotype [165]. Likewise, *Paenibacillus alvei* K165 was earlier demonstrated as an excellent BCA towards VWO, significantly decreasing the symptoms and severity of the disease on the susceptible cv. Amfissis in greenhouse experiments. Interestingly enough, the strain K165 was able to suppress VWO and reduce the pathogen biomass present in the tissues of “Amfissis” trees grown in naturally infested soils [166]. Similar results were obtained with *Bacillus velezensis* OEE1, a bacterium capable of significantly reducing the final mean disease severity index, percentage of dead plants, area under the disease progress curve, and number of MS in naturally infested soil [167].

Several fungi have been also identified as good BCA against *V. dahliae*. The non-pathogenic *Fusarium oxysporum* FO12 was able to reduce the viability of *V. dahliae* MS in naturally infested soils. This isolate also showed the ability to reduce symptoms in “Picual” plants under controlled conditions [168]. Likewise, treatments with conidial suspensions or chlamydospores of FO12 were effective in reducing both the inoculum density of *V. dahliae* in the soil and the VWO severity [169]. As for the biocontrol mechanism involved, the effect of secondary metabolites produced by FO12 against *V. dahliae* was investigated [169,170]. In vitro experiments indicated that the metabolites produced by FO12 had little effect on the inhibition of *V. dahliae* mycelial growth, suggesting that the antagonistic effect relies on the presence of FO12 alive and on a direct interaction with the pathogen [169]. In addition, the production of VOC by FO12 in the presence of *V. dahliae* has been reported. Several of these compounds have been previously involved in antifungal activity and biological control [170]. 

More fungal and bacterial BCA against *V. dahliae* can be added to this overview. For instance, isolates of *Blastobotrys* or *Arthrobacter* [144], *Achromobacter xylosoxidans* [171], *Mucor* spp., *Rhizopus* spp., or *Phoma* sp., [168] are examples of genera seldom investigated as BCA. It is worth mentioning that the particular case of *Phoma* sp. poses an interesting added value since it can be easily and directly applied to the aerial part of the olive plant [168]. Secondary metabolites produced by entomopathogenic fungi such as *Metarhizium brunneum* and *Beauveria bassiana* were reported by their antifungal activity against different plant pathogens, including *V. dahliae* [172,173]. The use of crude extracts and dialyzed fractions of both entomopathogenic fungi altered the mycelial growth, MS formation, and conidia germination in D and ND pathotypes of *V. dahliae* [172,173]. The effect of a number of environmental factors such as temperature, UV light exposure, and pH on the antifungal activity of the dialyzed fraction of *M. brunneum* was also investigated. The results showed that exposure to pH 7.5 and 8.5 for 24 h caused negative effects on its fungicidal activity, decreasing the inhibitory effect of the dialyzed fraction against *V. dahliae* mycelium [173]. This result confirms once again the need to perform evaluations of promising microorganisms also under field conditions, where climatic, environmental, and physical-chemical factors can show large ranges of variation.

The ubiquitous fungal genus *Trichoderma* includes more than 400 species. It has been described as BCA against many soil-borne plant pathogenic fungi. Some strains from diverse species have shown the ability to inhibit the growth of *V. dahliae*, but only a few of them have demonstrated the biocontrol of VWO. Indeed, the reduction in VWO symptoms and severity in the susceptible cv. Picual has been reported [168,174,175], as well as the inhibition of *V. dahliae* mycelial growth by crude extracts [172], extracellular compounds, or VOC [174,176]. By using fluorescently tagged derivatives of *T. harzianum* and *V. dahliae*, detailed imagery on the colonization process of both microorganisms, their interaction in olive root tissues, and the possible mechanisms of action of *Trichoderma* to control VWO have also been reported in recent years [175,177]. 

Despite the fact that arbuscular mycorrhizal fungi (AMF) have been investigated for its ability to control VWO (López-Escudero and Mercado-Blanco 2011, and references therein [16]), only a few published studies have reported progress on this topic during the last decade [178,179,180]. Plants infected with *V. dahliae* and pretreated with a native AMF consortium (Rhizolive) or with a *R. irregularis* (formerly *G. irregulare*) pure strain significantly reduced the VWO severity and the area under the disease progress compared with control plants in the cv. Haouzia [179]. In addition, pretreated olive plants with a new Rhizolive consortium enhanced the root mycorrhizal intensity in the presence of *V. dahliae* and decreased the percentage of the pathogen in the roots and stems of olive plants cv. Picholine Marocaine [180]. This effect has been attributed to a decrease in ROS accumulation and redox activities mediated by AMF compared with intact roots in contact with *V. dahliae* that showed an increase in ROS and nitric oxide. These results evidenced that olive root defense responses differed depending on whether the interaction takes place with a beneficial (AMF) or a deleterious (*V. dahliae*) fungus [178].

Recent trends in the biocontrol of trees (plants in general) diseases are focusing on the development and use of the consortia of beneficial microorganisms [13,181]. Thus, the combination of different bacteria is suggested to provide better biocontrol efficacy against VWO compared to the outcomes observed with individual treatments [165]. The joint application of some of the BCA mentioned in this review may serve to develop novel bioproducts. Thus, commercial biofungicides primarily composed of a mixture of different microorganisms such as *Ascophyllum nodosum, Bacillus subtilis* Y1336, *Streptomyces* spp., *Trichoderma* spp., or AMF are available. The application of different bio-formulations allowed the reduction of the incidence of VWO under controlled conditions [182]. Similarly, the effectiveness of a commercial biofungicide was tested in field conditions, and the tree recovery process was verified through spectral reflectance data [183]. 

### 6.6. The Olive Belowground Microbiota: Yet to Be Fully Uncovered and Understood

The olive belowground microbiota has been largely unknown until very recently. A comprehensive knowledge of the composition, structure, and functioning of the prokaryotic and fungal communities associated with olive roots will enhance our understanding of the health, development, and fitness of this tree crop. Moreover, under the holobiont conceptual framework, innovative perspectives can be foreseen in areas such as breeding for VWO resistance and the development of novel biocontrol tools [13,184]. Earlier, the olive associated microbiota was reported as an important reservoir of microorganism with potential as BCA against VWO [185]. 

Recently, the use of high-throughput sequencing allowed us to explore the composition and structure of microbial communities (fungi and bacteria) associated with olive cultivars from different geographical origins grown under the same climatic, agronomical, and pedological conditions [12]. Data revealed several interesting findings, making it possible to define a rather complete inventory of the rhizosphere and root endosphere microbiota (fungi and bacteria) associated with olive trees [12]. The results showed higher alfa diversity in the rhizosphere compared to that observed in the root endosphere. While previous studies revealed the importance of the geographical origin in building up the endophytic prokaryotic communities found in olive leaf tissues [185], our study concluded that the genotype, rather than geographical origin, is the main factor in shaping the olive belowground microbial communities. Moreover, the genotype is more determinant in the rhizosphere than in the root endosphere, and more crucial for the bacteriota than for the mycota [12]. The study also revealed that *Actinophytocola* was the most abundant genus inhabiting olive roots, followed by the genera *Streptomyces* and *Pseudonocardia*. Research efforts should be aimed at isolating and characterizing members of these relevant components of the olive belowground microbiota due to their potential role in contributing to the health and stress tolerance of olive trees. Concerning fungal communities, the results showed that a high percentage of sequences remained unclassified (10.7% in the root endosphere and 35.4% in the rhizosphere), which highlights the fact that the fungal diversity associated with olive roots is yet to be discovered. The prevalent classes present in the olive root endosphere were *Sordariomycetes* and *Eurotiomycetes*, while *Agaricomycetes*, *Eurotiomycetes,* and *Sordariomycetes* were the predominant classes in the rhizosphere. Future research efforts should also be aimed at isolating and characterizing members of these relevant components of the olive belowground mycota [12]. 

The results from the previous study enabled the characterization of the olive root-associated microbial communities in the absence of *V. dahliae* pressure. Yet, two important questions remained to be answered. On the one hand, it was uncertain whether the composition, structure, and functioning of these communities differ depending on the VWO tolerance level of olive cultivars. On the other hand, it was not known either what changes, if any, the root endosphere and rhizosphere microbial communities of olive cultivars with different VWO tolerances undergo upon inoculation with *V. dahliae*. Therefore, the objective of a very recent study aiming to address these questions was to assess whether the belowground microbial communities of cultivars Frantoio (VWO tolerant) and Picual (VWO susceptible) contribute to their differential disease susceptibility level [184]. Comparing the microbial communities of non-inoculated plants of each cultivar, some interesting differences were observed. At the phylum level, a higher abundance of beneficial genera was found in the root endosphere communities of the cv. Frantoio. In contrast, the “Picual” roots showed a major abundance of potential deleterious genera (e.g., *Fusarium*, *Macrophomina*, and *Rhizoctonia*). Consequently, the presence of harmful representatives of these genera could somehow increase the susceptibility of “Picual” roots to *V. dahliae* attacks. The introduction of *V. dahliae* did not provoke significant alterations either in the structure or in the functionality of the belowground microbial communities of any of the cultivars assessed. However, notable differences were found in their networks in response to the pathogen inoculation. Indeed, the analysis of the co-occurrence interactions revealed relevant topological alterations, mostly in the root endosphere after inoculation. Thus, the communities of the cv. Frantoio switched to a highly connected and low modularized network, while “Picual” communities showed sharply different behavior. Furthermore, the VWO-susceptible cv. Picual showed a higher increase in the number of negative interactions (e.g., competition and antagonism) than that observed in “Frantoio” plants. These results may explain, at least to some extent, the differential VWO susceptibility displayed by the tested olive cultivars [184]. Moreover, the triggering of a successful disease state in olive cultivars seems to be not only due to the complex global processes [42,75] taking place just between *V. dahliae* and olive, nor to the changes in co-occurrence interactions within the belowground fungal and bacterial communities upon interaction with the pathogen [184]. Recently, and by using a systems biology approach and metatranscriptomic analysis of the previously generated RNAseq data [43], a much more complex scenario has been unveiled. Indeed, additional components of the belowground biodiversity (i.e., amoebae, ciliates, and nematodes) can also play important roles, both in time and space, which still remain to be correctly interpreted [186].

### 6.7. Plant Extracts, Essential Oils, and Seaweeds 

Some plant extracts and essential oils can exert an effective control of plant pathogens [187,188] and insect pests [189,190]. These compounds are advantageous in terms of sustainability, mode of action, and low or null toxicity to be included in integrated management strategies [191]. However, regarding the control of Verticillium wilts in general and VWO in particular, only a few studies are available. For instance, the hydro-distilled essential oil from the leaves of *Juniperus thurifera* L. was able to inhibit the mycelial growth of different phytopathogenic fungi, including *V. dahliae* [192]. Similarly, the variable in vitro mycelial growth inhibition of *S. sclerotiorum*, *F. oxysporum,* and *V. dahliae* was observed with essential oils extracted from *Vaccinium myrtillus* L., *Laurus nobilis* L., and *Eucalyptus camaldulensis* Dehnh [193,194]. Finally, a study compared the antifungal effect of essential oils and plant extracts of *Mentha piperita* L., *Thymus vulgaris* L., and *Lavandula angustufolia* Mill., showing that essential oils provided better results for their ability to inhibit mycelial growth [195]. These results showed that a detailed knowledge of the chemical composition of the essential oils is needed to develop new effective formulas against plant pathogens. To the best of our knowledge, only one study has been performed to evaluate the effectiveness of plant extracts and essential oils against *V. dahliae* in olive plants [191]. The results showed that the essential oil from *Thymus* sp. 04 showed a remarkable inhibition of mycelial growth and MS viability, as well as a significant reduction in VWO. 

Seaweeds have a high polysaccharide content which is involved in early signaling processes through the activation of secondary metabolic pathways [196]. The use of seaweeds in the biological control of VWO has been seldom investigated. The effect of the application of potential elicitors like alginate and laminarin (brown algae), carrageenan (red algae), and ulvan (green algae) over phenylalanine ammonia-lyase (PAL) and lignin contents has been investigated. An increase in PAL activity was detected after ulvan or laminarin application in the twigs of olive cv. Picholine Marocaine. In addition, PAL is correlated with cell wall lignification and phenolic compound accumulation. In this respect, the lignin content increased significantly in twigs treated with alginate, carrageenan, or ulvan. Morevoer, twigs treated with alginate, laminarin, and ulvan experienced a 50% reduction in vascular browning [197].

## 7. Conclusions

What did we learn about VWO and its control during the last decade? This is the question proposed in the title of our review article. According to the main research efforts conducted and here summarized, we think that some answers can now be provided. First of all, VWO is still one of the most serious diseases affecting olive cultivation in many areas, particularly within the Mediterranean Basin. This is an unquestionable fact according to the available reports, even though a new emergent vascular pathogen for olive (i.e., *Xyllela fastidiosa*, causing the olive quick decline syndrome) is devastating specific olive growing areas [198]. Undoubtedly, the latter may represent an added serious biotic constraint for the future of this tree crop in the main olive growing countries [199], where VWO is (or can be) also present. Secondly, olive cropping systems have experienced significant changes during recent decades, aiming to improve mechanization, yield, and quality. However, the impact that this huge step forward for modernizing the traditional olive cultivation system has on traditional and emerging diseases/pests has seldom been evaluated. Moreover, the consequences of these modern practices on the health of soils and on the belowground olive-associated microbiome must not be overlooked. Therefore, research efforts should be conducted in this regard. Thirdly, the early and accurate detection of *V. dahliae* is crucial to successfully confront the disease. Sophisticated and accurate molecular diagnosis procedures are currently available, providing pathogen detection thresholds unimaginable a few years ago. The irruption of remote sensing technologies constitutes an air-borne defense strategy to control the disease at large scales, although efforts are still necessary to overcome potential misdetections with other biotic and abiotic stresses. Nevertheless, complementation with PCR-based approaches can overcome these problems. Fourthly, irrigation water has been demonstrated as an effective way to disperse infective propagules of *V. dahliae* over large distances. Therefore, the disinfection of irrigation water should be considered as a key control measure, among others traditionally proposed, to hinder the introduction of the pathogen to *V. dahliae*-free areas or to avoid the increase in propagules in endemic areas. 

**Figure 5:**
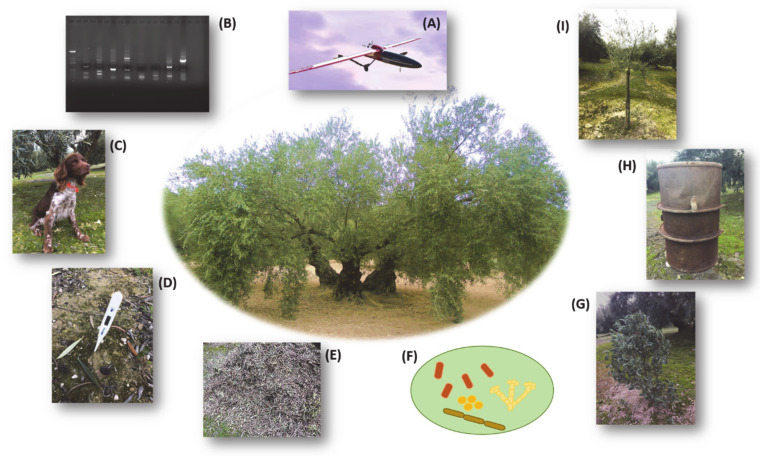
An idealized compilation of the available Verticillium wilt of olive control measures described in this review to be implemented within an integrated management strategy. (**A**) Use of spectral technologies coupled with drones or UAV for the early detection of the disease at a large scale (modified form an image kindly provided by Dr. J.A Jiménez Berni, IAS-CSIC, Córdoba); (**B**) identification based on molecular methods; (**C**) dogs trained for the detection of *Verticillium dahliae* volatile compounds; (**D**) heat treatments; (**E**) organic amendments; (**F**) biological control agents; (**G**) the use of plant extracts and essential oils; (**H**) water disinfection treatments; (**I**) new sources of tolerance/resistance.

The implementation of an integrated management strategy is the best way to deal with the disease (Figure 5). During the last decade, a considerable body of knowledge has been generated on issues such as screening, evaluation, and breeding for new genotypes/cultivars showing tolerance to VWO, their use being the most sustainable and economically plausible control measure. It must be emphasized that these efforts must be complemented with and assisted by a comprehensive knowledge of the races, pathotypes, and clonal lineages of *V. dahliae*. Likewise, a comprehensive understanding of the molecular and genetic mechanisms underlying the olive–*V. dahliae* interaction will be instrumental in VWO breeding programs. Thanks to the huge amount of data generated by the development and implementation of “-omics” approaches, the dialogue established between the host and the pathogen seems now to be better understood, as well as that taking place between them and the accompanying olive-associated microbial communities. Related to this, the contribution that the belowground microbiota seem to make in tolerance to VWO must not be neglected. Nevertheless, more efforts are needed to fully unveil the (i) complexity of this pathosystem, (ii) the multiplicity of factors influencing the onset and severity of VWO, and (iii) reasons for the inconsistency of control measures such as biocontrol, particularly at the field level. The disease must thus be confronted from a holistic perspective and within the holobiont conceptual framework.
